# The Emotional Temperament

**Published:** 1918-08-10

**Authors:** 


### HUMAN TEMPERAMENTS.

### The Emotional Temperament.

The whole feeling and tradition of English life is antagonistic to the emotional temperament, which is held nowhere in such disrepute and disfavour as in this country. We regard the facile and ready display of signs of emotion as a feminine characteristic, unworthy and derogatory in a man; and if, as there is good reason to suppose, that nervous system is the most highly developed and most highly organised in which the control and inhibition of the higher centres over the lower are the most complete, there is much to be said for the English view. When we say English, we include also the lowland Scotch, who are of the same original stock, which is in them less mixed by successive infusions of alien blood; and we exclude the Celtic fringes, in which the same tradition does not obtain, but in which, on the contrary, the emotional temperament is highly developed. Thackeray draws for us the picture of a perfect servant, who waited punctually at table, cleared everything away, and when all his duties were performed asked permission to go home, on the ground that he had received information that his house was on fire. Thackeray does not draw the inference, but we know quite well that the man was an Englishman born and bred. He was not Scotch, for who ever heard of a Scotch butler, at any rate out of Scotland? And it is to be presumed that if there were any Scottish butlers they would be found in England. Nor was he Irish or Welsh, for apart from the rarity of butlers of either nationality in England, an Irishman or a Welshman would have rushed into the dining-room immediately on receipt of the news, would have explained with much gesticulation and little coherency the nature of the disaster, and have rushed out again to the scene of it, leaving the dinner and the dinner-things to take care of themselves.

It is no part of the task of these papers to apportion praise or blame. They are purely descriptive, and in no degree didactic. It is open to any reader to decide for himself which course of conduct he considers more proper in the circumstances; and there is no doubt that while that described by Thackeray will be generally approved in England, with an approval that increases as we travel northward, it will be regarded in the Celtic fringes as incomprehensible, and by no means to be admired.

The name of the Emotional Temperament describes it, at least in part. Its possessors express their emotions readily and strongly. There is no reason to think that they feel more deeply or more acutely than the unemotional, but whatever they feel they must express. They must express it at once, and strongly, often, it seems, more strongly than the feeling needs or warrants. They are deficient in reticence, in self-restraint, in self-control. Their emotions are near the surface, and are constantly breaking through the surface and becoming plain to the bystander. The expression of their emotions is set off by a hair trigger, and often, indeed, goes off at half-cock. They are prone to express emotions, and to express not only what they feel, but often more than they feel.

Emotion is expressed in various ways; in facial expression, attitude, demeanour and gesture, in voice, articulation, and speech; and in all these ways the emotional person expresses himself, often with exaggeration.

The common mode of expressing the emotions of depression is by weeping, and hence emotional persons are readily moved to tears. They weep on small occasion, freely and unrestrainedly, loudly and demonstratively, with no attempt at repression. Saul, an emotional man, belonging to an emotional race, "lifted up his voice and wept." The description is admirably expressive. It displays the man's entire lack of restraint, his indifference to the presence of spectators. Nay, the presence of spectators was probably a stimulant to him to give rein to his feeling. An Englishman may weep upon occasion, but the occasions are very rare, and in no case would he lift up his voice and weep unrestrainedly. He would be ashamed, and would do his best to repress and hide the expression.

Emotion is expressed mainly in speech and in action, and in both ways the emotional person expresses his emotions readily, with freedom, and with exaggeration, often running into extravagance. In speech, he is prone to the use of strong expressions and superlatives. Emotional persons are perhaps more numerous than they used to be, and form a larger proportion of the population; at any rate, the misuse and degradation of strong and superlative expressions is become much more frequent of late years, possibly because of the permeation of the country by the Celtic fringes. Such words as awful, perfectly, infinitely, absolutely, frightfully, and so on, have been so misused and vulgarised that they have lost their intensitive meaning, and have almost lost their meaning altogether. The emotional person uses them perpetually. He piles Pelion on Ossa. He would drink up Esil, eat a crocodile, worship mighty Mumbo Jumbo in the Mountains of the Moon; but his emotion evaporates in talk, and his performance falls far short of his declared intention. He is apt to say more than he means, and much more than he will stick to.

For the emotional person is by nature untruthful. He is untruthful in both ways—that is, he says carelessly and unthinkingly what is not in accordance with fact, not recognising or not admitting the desirability of truthfulness, not caring whether what he says is true or not; and besides this, and no doubt on account and by reason of this, he often lies in the second of Dr. Johnson's senses. He lies, and he knows he lies.

**Human Temperaments**—*(continued)*.

His assertions are, like all his expressions, exaggerated; and they are variable. What he has said one minute he will unsay the next. Self-contradiction has no terrors for him. He cares little for being detected in an untruth, for he has no proper appreciation of the value and beauty of truth. He is constitutionally inaccurate. You cannot believe a word he says.

But his use of emotional speech is so frequent that he is fluent. He gains by constant practice an easy mastery over words and phrases. He is always fluent of speech, and his fluency often rises into eloquence. The emotional races, the Irish especially, are renowned for their eloquence. We are far from saying that eloquence is restricted to the emotional temperament. Were we to say so, the single case of John Bright would be enough to refute us; but undoubtedly eloquence and even oratory are frequent among the emotional, infrequent among the reticent, the self-controlled, the self-contained. The emotional orator easily becomes a demagogue; and the majority of demagogues have been of this temperament. Their appetite for expression grows with their opportunities for expression, and when they are on their feet addressing a sympathetic audience, they are apt to be carried away, to say far more than they intended to say, to advance to positions they never meant to occupy. I[illegible] emotional orator is addressing an emotional audience, the consequences cannot be foretold, but it is certain that they will be momentous. We see the result in revival meetings, in which the effect of emotional eloquence on an emotional audience is heard in shouts and ejaculations, and seen in weeping, in swooning, and even in convulsions among the audience. We see it also in the terrible destruction wrought by a mob of emotional malcontents addressed by an emotional demagogue.

In action, the emotional person is impulsive. Wanting as he is in self-restraint, he does not wait to act until he has balanced the advantages and disadvantages of action. He is wanting in circumspection and deliberation. The path from feeling to action is short-circuited. As the emotional cannot bear pain without howling, so they cannot bear suspense, which is a kind of pain, without a struggle to relieve it. They cannot wait. Accustomed to express their emotion as soon as it is felt, they must express it so in action as well as in words, if it is susceptible of expression in action. They want results immediately, and they think that, even in the most complicated affairs, results may be attained immediately. They rush direct for their goal, not recognising that in complicated affairs, and especially in social affairs, direct action is usually the direct route to failure. They are too impatient to think out in detail an elaborate scheme requiring time to bring it to maturity, and needing scrupulous attention to detail to ensure its success, so they rush at some crude project, and are content to take credit for good intentions, and to lay the blame of failure upon those who have to execute an impossible task.

Emotional people act upon impulse. This does not necessarily mean that their action is sudden or abrupt. An abrupt action may have been well considered and thought out beforehand, and considered and well-thought-out acts are the opposite of impulsive. An impulsive act may have been long determined on, and postponed until opportunity presented itself. The mark of impulsive action is not suddenness or abruptness, but want of due estimation of the advantages and disadvantages of the act. The emotional person is impatient. His emotion burns to express itself in action. He is long accustomed to let his emotion boil over in action, and he cannot wait to consider, so his action is immediate and direct. He will rather jump out of the window than go down the staircase; he will rather splash through the pond than go round by the bank; and the consequence is that in the first case he is apt to break his leg, and in the second to get out of his depth. The emotional person is, in fact, very generally out of his depth. With a light heart and an ignorance of consequences he lets loose forces that he cannot control or direct. He launches crude and undigested schemes that produce all kinds of results except that which he intended. The most dangerous person in the world, the fertile source of incalculable and innumerable mischiefs, disasters, and injustices, is the well-intentioned enthusiast who is also an emotional person.

Enthusiasm is the great motive power of humanity, and without it no great unselfish project was ever carried through. Enthusiasm held in hand by self-restraint and guided by sound judgment has given us every great discovery, every difficult invention, every new religion, almost every great benefit that humanity has received, from geometry to porcelain, from the theory of gravitation to the steam-engine, from natural selection to electricity; but the unrestrained and unguided enthusiasm of the emotional temperament has been responsible for most of the great disasters from which the human race has suffered. The emotional enthusiasm of St. Bernard and of Peter the Hermit sent many hundreds of thousands of men and women to their death in the Balkan peninsula, before Constantinople, and in the sands of Palestine; the emotional enthusiasm of Torquemada produced the horrors of the Inquisition; the emotional enthusiasm of Innocent VIII. initiated the horrors of the witch mania; the emotional enthusiasm of John Law floated the Mississippi Scheme; the emotional enthusiasm of Harley blew up the South Sea Bubble; and in our day emotional enthusiasm has produced the ludicrous fiasco of the Increment Tax and the chaos of the Insurance Act.

